# Development and content validation of the Satisfaction and Experience Questionnaire for Granulocyte Colony-Stimulating Factor (SEQ-G-CSF)

**DOI:** 10.1186/s41687-020-00277-8

**Published:** 2021-01-18

**Authors:** Aylin Yucel, Anne Skalicky, Olabimpe Ruth Eseyin, Emre Yucel, Rajesh Belani, Mark Bensink

**Affiliations:** 1 Amgen Inc., One Amgen Center Drive, Thousand Oaks, CA USA; 2 Evidera, Seattle, WA USA; 3 Evidera, Bethesda, MD USA

**Keywords:** Myelosuppressive chemotherapy, Febrile neutropenia, G-CSF, On-body injector, OBI, Content validation, Satisfaction and Experience Questionnaire for Granulocyte Colony-Stimulating Factor, SEQ-G-CSF

## Abstract

**Background:**

Several options for granulocyte colony-stimulating factor (G-CSF) prophylaxis of chemotherapy-induced febrile neutropenia are available to patients worldwide. We have developed a novel patient-reported outcome measure, the Satisfaction and Experience Questionnaire for G-CSF (SEQ-G-CSF), to help understand patients’ perspectives of and satisfaction with different G-CSF options.

**Results:**

Three oncology nurses and 40 adult oncology patients in the United States were enrolled and participated in focus group discussions to develop and refine the SEQ-G-CSF. Nurses had ≥ 5 years of experience treating oncology patients and were currently involved in the management of oncology patients receiving G-CSF prophylaxis. The patients had breast cancer, lung cancer, non-Hodgkin lymphoma, or prostate cancer (10 patients in each group) and were receiving G-CSF prophylaxis via injection or the on-body injector (OBI) device. The preliminary SEQ-G-CSF contained an item relevance questionnaire and three SEQ modules (sociodemographic, medical history, and G-CSF–related healthcare characteristics questionnaires). Twenty-one patients (53% of total sample size) discussed their experience and satisfaction with G-CSF. Their most common experiences were G-CSF effectiveness, convenience and benefits of the OBI, and relationships with healthcare providers. Side effects and having to undergo additional treatment were also reported. Satisfaction with aspects of G-CSF included the OBI and effectiveness of G-CSF treatment; dissatisfaction included inconvenience (having to return to the clinic the next day and administration of the injection) and the insurance approval process. The SEQ-G-CSF was finalized after three rounds of cognitive interviews and includes five domains related to general satisfaction (one item), treatment burden (four items), travel burden (two items), time burden (four items), and treatment compliance (two items).

**Conclusions:**

The SEQ-G-CSF is a novel instrument that quantifies a patient’s experience and satisfaction with different G-CSF options using 13 easy-to-understand items. This study provides evidence for the content validity of SEQ-G-CSF. Although further psychometric testing is required, the SEQ-G-CSF may be a useful addition to clinical trials, observational studies, and clinical practice.

**Supplementary Information:**

The online version contains supplementary material available at 10.1186/s41687-020-00277-8.

## Plain English summary

### What is the key problem/issue/question this manuscript addresses?

One of the side effects of chemotherapy is febrile neutropenia (FN), a condition associated with fever and low numbers of certain white blood cells. Granulocyte colony-stimulating factors (G-CSF) are recommended as a preventive treatment for chemotherapy-induced FN for many types of cancer. With different types of G-CSFs as well as different administration modes of G-CSFs, and currently no available valid method to quantify patients’ experience with G-CSF, the study objective was to develop an instrument to assess the chemotherapy patient’s satisfaction and experience with these different G-CSF treatment options.

### Why is this study needed?

The recommendation of G-CSF use by physicians and hospitals varies based on preference, and there is currently no valid method to quantify patients’ experience with G-CSF. Thus, it is important to have an established metric for measuring satisfaction in patients who have received G-CSF treatment, to help understand their perspective.

### What is the main point of your study?

We developed a novel questionnaire (SEQ-G-CSF) to quantify a patient’s experience and satisfaction with different G-CSF options, using 13 easy-to-understand items. The questionnaire was developed with input from patients and nurses to ensure that it fully captures their experience.

### Provide a brief overview of your results and what they mean

This study has provided evidence of content validity for the SEQ-G-CSF, and our results indicate that patients understand the items in the questionnaire. While additional testing of other forms of validity and reliability is required, the SEQ-G-CSF may be a useful addition to clinical trials, observational studies, and clinical practice. Results of studies including this new tool will provide information to help patients and providers make an educated decision on their treatment, when there are multiple options for preventing FN.

## Background

Chemotherapy-induced febrile neutropenia (FN) is associated with negative outcomes such as extended hospital stays, higher medical costs, dose reductions, dose delays, poorer treatment response, and higher risk of mortality [1]. Current guidelines recommend the use of granulocyte colony-stimulating factor (G-CSF) as prophylaxis of chemotherapy-induced febrile neutropenia when the risk of febrile neutropenia is ≥ 20% and to consider its use when the risk is 10%–20% [2–5]. Some of the most common tumor types that utilize the G-CSF particularly as primary prophylaxis are breast cancer, lung cancer, non-Hodgkin lymphoma, and prostate cancer [6–9] with an average of about 20% across various metastatic cancers in the US [10]. In the US, the prevalence of patients with metastatic cancer (having high risk of FN at > 20% and intermediate risk of FN at 10–20%) requiring G-CSF is about 45% [11]. G-CSFs are either short- or long-acting, and when administered as multiple injections per chemotherapy cycle (eg, filgrastim) or as a single injection administered once per chemotherapy cycle (eg, pegfilgrastim), they reduce the risk of febrile neutropenia and infection-related mortality [12–15].

Although pegfilgrastim is administered once per chemotherapy cycle, offering increased convenience to patients and caregivers, it should be administered the day after chemotherapy [16]. It is often inconvenient for patients to return to the clinic; therefore, the pegfilgrastim on-body injector (OBI) was developed to deliver pegfilgrastim approximately 27 h after its application [17, 18].

Factors that could influence use of G-CSF include clinical guidelines, hospital protocol, inclusion of G-CSF on drug formularies and physician and patient preference [19] as well as patient experience. Despite the availability of several options for prophylaxis of chemotherapy-induced febrile neutropenia, there is currently no valid method to quantify patients’ experience with G-CSF. We have developed a novel patient-reported outcome (PRO) measure, the Satisfaction and Experience Questionnaire for G-CSF (SEQ-G-CSF), to help understand patients’ perspectives of and satisfaction with different G-CSF options. Results of studies using this novel PRO measure may provide useful information on patient treatment experience to help with patient and clinician decision making.

## Methods

**Fig. 1:**
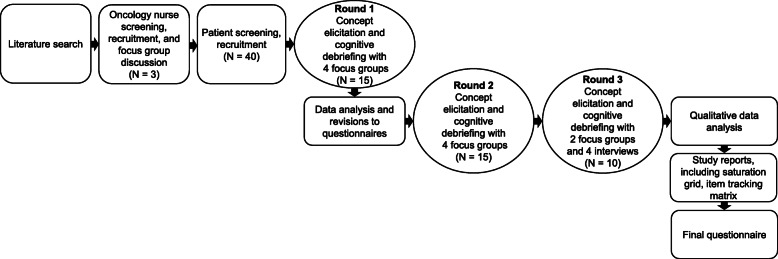
Project flow

The SEQ-G-CSF instrument was developed using an iterative process (Fig. 1). The concept identification stage (identification of applicable outcome measures from the literature) and the concept elicitation and confirmation stage (focus group discussions with oncology nurses and oncology patients) informed the content of the initial version of the SEQ-G-CSF.

### Stage I concept identification: development and refinement of the SEQ-G-CSF

Prior to initiation of the study, we were unable to identify a fit-for-purpose PRO measure relevant for understanding patients’ experience and satisfaction with different G-CSF options. We therefore developed a de novo measure based on desk research with a preliminary conceptual framework that adapted relevant concepts from several existing treatment satisfaction tools [20–23]. Preliminary drafts of the SEQ-G-CSF were modified by the study staff based on accepted best practices and guidance for PRO development [24–27]. All pertinent feedback or recommendations were considered, including a Flesch-Kincaid readability assessment, to reduce the grade-level of items [28]; the conceptual framework and related items were revised accordingly.

### Nurse focus groups

Oncology nurses were screened for eligibility and recruited through a third-party recruitment vendor to participate in focus group discussions conducted through an online platform. Eligible oncology nurses had ≥ 5 years of experience with treating oncology patients and were currently involved in the management of oncology patients receiving G-CSF prophylaxis. The 60-min nurse focus group discussion took place via a webcam link. A semi-structured, standardized discussion guide was used in the focus group discussions with oncology nurses. The purpose of the focus groups was to assess the patient experience and satisfaction with G-CSF. Topics included their background and experience as well as their assessment of patient experience and satisfaction with oncology treatment and G-CSF prophylaxis (Table 1). Nurses also answered several closed- and open-ended questions using polling software. Nurse focus groups discussions were conducted before patient focus group discussions, and findings from the nurse discussions informed development of the patient concept elicitation discussion guides.

**Table 1:** Concept elicitation and cognitive interview discussion topics

Examples of nurse discussion topics	• What are cancer patients’ expectation of chemotherapy?
	• How do cancer patients balance chemotherapy treatment satisfaction and treatment effectiveness in light of experiences of side effects?
	• What are patient expectations of G-CSF primary prophylaxis treatment?
	• What are patient experiences of G-CSF primary prophylaxis?
	• What specific challenges or burdens do patients face with each mode of administration (eg, intravenous, subcutaneous, OBI)?
Examples of patient discussion topics	• What were the most important factors that determine whether your chemotherapy treatment experience was positive?
	• How do you know that your G-CSF treatment was working well for you?
	• What were the most important factors that determine whether your G-CSF treatment experience was positive?
	• What are the main advantages/disadvantages of the OBI treatment?
SEQ discussion topics	• What are your first impressions of this questionnaire? What did you think about the length of the questionnaire? Would you remove or change any questions to the questionnaire?
	• What did the instructions ask you to do? Would you change anything about the instructions?
	• What does [item] mean to you? What were you thinking about when you selected your answer?
	• Which answer did you choose? What do you think of the response options?
	• How could the question be made clearer?

*Abbreviations*: *G-CSF* granulocyte colony-stimulating factor, *OBI* on-body injector, *SEQ* Satisfaction and Experience Questionnaire

### Patient focus groups

Breast cancer, lung cancer, non-Hodgkin lymphoma, and prostate cancer patients were screened for eligibility and recruited through a third-party recruitment vendor to participate in focus group discussions on an online platform.

Oncology patients were required to submit proof of a physician’s verification of cancer diagnosis and chemotherapy medication. Other eligibility criteria included: patients aged ≥ 18 years old with a self-report of breast cancer, lung cancer, non-Hodgkin lymphoma, or prostate cancer as well as current or recent (< 6 months from screening date) chemotherapy and prophylaxis with G-CSF. In addition, patients were required to be US residents, have access to the internet, have sufficient proficiency in the English language to complete the consent process and participate in focus group discussions, and be willing to be audio-recorded during discussions.

Based on the types of concepts being explored, it was estimated that a minimum of 10 patients were required per group to achieve saturation [29]. Quota sampling was utilized to recruit 40 patients from four cancer groups (breast cancer, lung cancer, non-Hodgkin lymphoma, and prostate cancer; *n* = 10 in each group) and two subgroups (OBI-naive: patients who had never used the OBI; OBI-experienced: patients who had used or are currently using the OBI; *n* = 20 in each group). The target quotas were to recruit ten patients per cancer type: five who were OBI-naive and five who were OBI-experienced. The goal was to conduct up to three rounds of focus groups; a minimum of two to three focus groups per round.

The study protocol was reviewed and approved by Quorum Review Institutional Review Board (approval number 33649), and all participants provided their written informed consent before the start of each interview discussion.

### Patient experience and satisfaction with G-CSF: concept elicitation

The objective of the concept elicitation portion of the patient focus group was to elicit patients’ experience with G-CSF prophylaxis (Table 1). Semi-structured discussion guides were developed with specific questions for OBI-naive and OBI-experienced patient groups. A Microsoft PowerPoint presentation based on the semi-structured patient discussion guide was developed for use during focus group discussions and telephone interviews. The presentation allowed patients to view the treatment satisfaction and experience topics. Individual interviews took place when patients were not available for group discussions.

Focus group discussions took place via a webcam link; 90 min for Round 1 and 2 participants and 60 min for Round 3 and individual interviews. Each focus group discussion was audio- and video-recorded, and transcripts from audio data were de-identified and coded. All participants were compensated for their time based on an assessment of the fair market value and length of focus group discussion.

Based on results from round 1 concept elicitation focus group discussions, the initial version of the SEQ-G-CSF was revised, and additional hybrid concept elicitation and cognitive interview focus groups were planned as needed to support attainment of concept saturation.

### Content confirmation of SEQ-G-CSF: cognitive interview

The objective of the cognitive interview portion of the patient focus group was to elicit patients’ experience with G-CSF prophylaxis and assess comprehension whether the instructions, items, response options, and terminology in the SEQ-G-CSF were understandable and relevant (Table 1). The initial version of the SEQ-G-CSF was shared with oncology patients during each of the three rounds of focus groups. Patients completed draft SEQ-G-CSF and then participated in a group cognitive interview. The PowerPoint presentation, which included the initial SEQ-G-CSF items and response options alongside alternative items and response options, was used during the cognitive interview portion for patient review and discussion.

After the cognitive interview portion, patients completed three additional SEQ modules, including an SEQ-G-CSF item relevance questionnaire (Supplementary Materials). The item relevance questionnaire obtained relevance ratings for each item of the SEQ-G-CSF. Patients rated the relevance of each SEQ-G-CSF item based on their medical condition and treatment, and responses were recorded on a five-point verbal rating scale. The three SEQ modules were used for collecting descriptive information: sociodemographic (SEQ module 1), medical history (SEQ module 2), and G-CSF-related healthcare characteristics (SEQ module 3) questionnaires. Patients provided feedback on the SEQ-G-CSF.

### Data analysis

Saturation was defined as the point at which no substantially new themes, descriptions of a concept, or terms were introduced as additional rounds of focus group discussions were being conducted. The concepts arising from the concept elicitation sessions were evaluated for saturation as the discussions were being conducted. No further additional interviews were considered if saturation was achieved.

During focus group discussions, structured counts were taken for each treatment experience and satisfaction concept to document saturation using a saturation grid that displayed the first mention of emerging concepts. For the concept elicitation portion, a coding framework was developed related to the meaning of treatment experience and satisfaction. The preliminary conceptual framework was imported into ATLAS.ti v8 to code the concept elicitation portion of the focus group transcripts. A content analysis approach was used to analyze and interpret the concept elicitation–coded data [30]. For the cognitive interview portion, a coding framework was developed to document focus group discussions specific to the SEQ-G-CSF instructions, original items and response options, alternative items and response options, and any spontaneous feedback related to missing concepts or suggested wording changes.

Quantitative data from case report forms were descriptive (n, mean, standard deviation, and/or frequency); analyses were done using SAS version 9.4 [31].

## Results

### Nurse characteristics

Four oncology nurses were screened and were eligible to participate in the focus group discussions; however, one nurse was unable to participate on the scheduled date. On average, each of the three nurses who participated in the study saw 118 oncology patients per week across the four cancer groups, had experience administering G-CSF (filgrastim and pegfilgrastim, an average of 13 patients per week), and had provided the OBI to patients in each of the four cancer groups (an average of six patients per week).

### Patient characteristics

A total of 40 patients who were receiving G-CSF, 19 OBI-experienced and 21 OBI-naive, were enrolled. Between December 06, 2018, and January 17, 2019, 10 focus group discussions were conducted with 36 patients, and individual interviews were conducted with four patients. All 40 patients completed the questionnaires and participated in the cognitive interview portion; data were collected from three rounds of focus groups. Fifty-three percent of the participants (*n* = 21/40) participated in the concept elicitation portion of the focus groups.

Two patients with prostate cancer did not return all the required study forms, and they were not included in all quantitative analyses; they did, however, contribute qualitative data and were included in those analyses. Patient characteristics and medical histories are shown in Table 2.

**Table 2:** Patient characteristics and medical history

Characteristics	Breast Cancer ***N*** = 10	Lung Cancer ***N*** = 10	Non-Hodgkin Lymphoma ***N*** = 10	Prostate Cancer^**a**^ ***N*** = 10	Total ***N*** = 40
Gender, n (%)					
Male	0 (0)	6 (60)	4 (40)	10 (100)	20 (50)
Female	10 (100)	4 (40)	6 (60)	0 (0)	20 (50)
Age					
Mean (SD) years	47.0 (9.3)	52.3 (17.1)	52.8 (14.0)	52.6 (5.7)	51.1 (12.4)
Median (range) years	51 (30–55)	51 (34–90)	52 (30–76)	51 (47–64)	52 (30–90)
Ethnicity, n (%)					
Not Hispanic or Latino	9 (90)	10 (100)	10 (100)	7 (70)	36 (95)
Missing	1 (10)	0 (0)	0 (0)	3 (30)	4 (10)
Racial background, n (%)					
Asian	0 (0)	2 (20)	0 (0)	1 (10)	3 (8)
Black or African American	4 (40)	0 (0)	0 (0)	0 (0)	4 (10)
White	6 (60)	8 (80)	10 (100)	5 (50)	29 (73)
Missing	0 (0)	0 (0)	0 (0)	4 (40)	4 (10)
Employment status, n (%)^b^					
Employed, full-time	5 (50)	5 (50)	5 (50)	6 (60)	21 (53)
Employed, part-time	3 (30)	2 (20)	3 (30)	0 (0)	8 (20)
Homemaker	1 (10)	0 (0)	1 (10)	0 (0)	2 (5)
Unemployed	0 (0)	1 (10)	0 (0)	0 (0)	1 (3)
Retired	0 (0)	0 (0)	1 (10)	0 (0)	1 (3)
Disabled	1 (10)	2 (20)	0 (0)	0 (0)	3 (8)
Missing	0 (0)	0 (0)	0 (0)	4 (40)	4 (10)
Highest level of education, n (%)^b^					
Secondary/high school	0 (0)	1 (10)	1 (10)	0 (0)	2 (5)
Some college	2 (20)	0 (0)	1 (10)	1 (10)	4 (10)
College degree	7 (70)	4 (40)	5 (50)	4 (40)	20 (50)
Postgraduate degree	1 (10)	5 (50)	3 (30)	1 (10)	10 (25)
Bachelor of Science/Bachelor of Arts	0 (0)	0 (0)	1 (10)	0 (0)	1 (3)
Missing	0 (0)	0 (0)	1 (10)	4 (40)	5 (13)
Insurance status, n (%)					
Insured, minimal out-of-pocket costs	6 (60)	9 (90)	8 (80)	4 (40)	27 (68)
Insured, significant out-of-pocket costs	4 (40)	1 (10)	2 (20)	2 (20)	9 (23)
Missing	0 (0)	0 (0)	0 (0)	4 (40)	4 (10)
Time since cancer diagnosis, n (%)					
< 6 months	3 (30)	0 (0)	2 (20)	2 (20)	7 (18)
6 months to 1 year	4 (40)	6 (60)	1 (10)	0 (0)	11 (28)
1–2 years	1 (10)	2 (20)	4 (40)	1 (10)	8 (20)
2–3 years	2 (20)	1 (10)	0 (0)	3 (30)	6 (15)
3+ years	0 (0)	1 (10)	3 (30)	2 (20)	6 (15)
Missing	0 (0)	0 (0)	0 (0)	2 (20)	2 (5)
Metastatic cancer	2 (20)	6 (60)	4 (40)	4 (40)	16 (40)
Currently receiving chemotherapy	8 (80)	6 (60)	4 (40)	3 (30)	21 (53)
Last chemotherapy treatment					
< 6 months	10 (100)	9 (90)	7 (70)	7 (70)	33 (83)
6 months to 1 year	0 (0)	0 (0)	3 (30)	1 (10)	4 (10)
1–2 years	0 (0)	1 (10)	0 (0)	0 (0)	1 (3)
Missing	0 (0)	0 (0)	0 (0)	2 (20)	2 (5)
Recently received or currently receiving G-CSF prophylaxis					
Currently receiving	7 (70)	3 (30)	1 (10)	1 (10)	13 (33)
Recently completed	3 (30)	7 (70)	9 (90)	7 (70)	13 (33)
Missing	0 (0)	0 (0)	0 (0)	2 (20)	2 (5)
Type of G-CSF prophylaxis^b^					
Pegfilgrastim	5 (50)	4 (40)	5 (50)	5 (50)	19 (48)
Filgrastim	5 (50)	6 (60)	5 (50)	2 (20)	18 (45)
Missing	0 (0)	0 (0)	0 (0)	2 (20)	2 (5)
Last G-CSF treatment, n (%)					
< 6 months	7 (70)	9 (90)	7 (70)	7 (70)	30 (75)
6 months to 1 year	1 (10)	1 (10)	2 (20)	1 (10)	5 (13)
1–2 years	0 (0)	0 (0)	1 (10)	0 (0)	1 (3)
Missing	2 (20)	0 (0)	0 (0)	2 (20)	4 (10)
Route of administration of G-CSF prophylaxis^b^					
Subcutaneous	5 (50)	1 (10)	6 (60)	3 (30)	15 (38)
Intravenous	2 (20)	5 (50)	2 (20)	2 (20)	11 (28)
OBI	3 (30)	4 (40)	2 (20)	3 (30)	12 (30)
Missing	0 (0)	0 (0)	0 (0)	2 (20)	2 (5)
Have you ever used an OBI for G-CSF treatment?					
Yes	4 (40)	7 (70)	4 (40)	7 (70)	22 (55)
Comorbidities					
No other health condition	10 (100)	10 (100)	9 (90)	8 (80)	37 (93)
Cardiovascular disease	0 (0)	0 (0)	1 (10)	0 (0)	1 (3)
Missing	0 (0)	0 (0)	0 (0)	2 (20)	2 (5)
Health status within the past week					
Excellent	2 (20)	0 (0)	3 (30)	1 (10)	6 (15)
Very good	0 (0)	1 (10)	3 (30)	2 (20)	6 (15)
Good	4 (40)	5 (50)	3 (30)	3 (30)	15 (38)
Fair	4 (40)	4 (40)	1 (10)	2 (20)	11 (28)
Missing	0 (0)	0 (0)	0 (0)	2 (20)	2 (5)

*Abbreviations*: *G-CSF* granulocyte colony-stimulating factor, *NHL* non-Hodgkin lymphoma, *OBI* on-body injector, *SD* standard deviation

^a^Two of the ten participants with prostate cancer did not return their study forms

^b^Data are not mutually exclusive

The proportion of male and female patients was the same, and the median age was 52 (range: 30–90) years. Overall, 40% of patients had metastatic cancer, 83% had completed chemotherapy within 6 months of enrollment, 75% had received their last G-CSF treatment within 6 months of enrollment, and 48% had received pegfilgrastim for febrile neutropenia prophylaxis.

### Nurse concept elicitation

Oncology nurses described patients’ satisfaction and tolerability with chemotherapy and G-CSF treatment based on their own clinical practice experience. Nurses reported that patients receiving chemotherapy and G-CSF derive satisfaction from experiencing a treatment response (an indication that the treatment is working), minimal hospital stays and co-pays, and good communication with their cancer care team. Nurses also stated that patients tend to tolerate treatment side effects if they see a treatment response or if their quality of life is not significantly impacted.

### Patient concept elicitation

The concept elicitation portion of the patient focus groups identified patients’ experience and satisfaction with chemotherapy and G-CSF prophylaxis, including side effects, treatment effect, and the treatment process and setting.

#### Patient experience and satisfaction with chemotherapy

Eighteen patients (45% of total sample size) discussed their experience and satisfaction with chemotherapy. The most common positive aspects related to patients’ experience with chemotherapy were treatment effectiveness (50%), relationships with healthcare providers (39%), and having supportive relationships (22%). The most common negative aspects were side effects of chemotherapy (56%); emotional burden, including fear and feelings of vulnerability (33%); time burden due to frequent treatment visits, follow-up visits, and time spent waiting for and receiving treatment (33%); concerns over drug safety (33%); and treatment burden (22%).

Satisfaction with aspects of chemotherapy included positive interactions with healthcare providers (33%), treatment effectiveness (28%), minimal time burden (17%), and having insurance and minimal paperwork (11%). Dissatisfaction with aspects of chemotherapy were health outcomes after chemotherapy (33%), disease course (22%), treatment burden (22%), and side effects (17%).

#### Patient experience and satisfaction with G-CSF

Twenty-one patients (53% of total sample size) discussed their experience and satisfaction with G-CSF. The most common positive experiences were G-CSF effectiveness (90%), convenience (43%, only reported by patients who had used the OBI), relationships with healthcare providers (38%), and benefits of the OBI (29%), including convenience, ease of use, available support, and reduced travel and time burden (Table 3). The most common negative experiences were side effects (33%, lethargy and fatigue) and having to undergo additional treatment (19%, more medication). Additional concerns with G-CSF were injection discomfort (10%), sleep interruption (10%), increased emotional stress (5%), G-CSF not being a cure (5%), inadequate G-CSF knowledge (5%), missing work (5%), and insufficient monitoring for subcutaneous G-CSF users (5%).

**Table 3:** Patient experience and satisfaction with G-CSF^a^

	Patients
**Concepts related to G-CSF experience, n (%)**	***N*** **= 21**
G-CSF effectiveness	19 (90)
Convenience	9 (43)
Relationship with healthcare provider	8 (38)
Side effects	7 (33)
OBI benefits	6 (29)
More medication	4 (19)
Self-efficacy	2 (10)
Injection discomfort	2 (10)
Sleep interruption	2 (10)
Increased emotional stress	1 (5)
G-CSF not being a cure	1 (5)
Inadequate G-CSF knowledge	1 (5)
Missing work	1 (5)
Insufficient monitoring for subcutaneous G-CSF users	1 (5)
**Concepts related to G-CSF satisfaction/dissatisfaction, n (%)**	***N*** **= 17**
OBI^a^	13 (76)
G-CSF effectiveness	8 (47)
Inconvenience	4 (24)
Dose regulation	3 (18)

*Abbreviations*: *G-CSF* granulocyte colony-stimulating factor, *OBI* on-body injector

^a^OBI users only

Satisfaction with aspects of G-CSF were the OBI (13%, among those who used the device) and effectiveness of G-CSF treatment (47%). Dissatisfaction with aspects of G-CSF were inconvenience (24%, having to return to the clinic the next day and administration of the injection) and dose regulation (14%, not seeing dramatic effects of the G-CSF treatment or not being weaned off their current dose) (Table 3).

### SEQ-G-CSF cognitive interview results

A total of 10 focus group discussions with 36 patients and individual interviews with four patients were conducted: four focus group discussions in round 1 (*n* = 15), four focus group discussions in round 2 (*n* = 15), and two focus group discussions (*n* = 6) and four individual interviews in round 3. Of the 40 patients who participated, 65% provided their overall impression of the SEQ-G-CSF item relevance questionnaire, 20% stated that questions should be removed, 18% stated that questions should be changed, 13% stated that the length of the questionnaire was appropriate, and 13% stated that additional questions were needed.

**Fig. 2:**
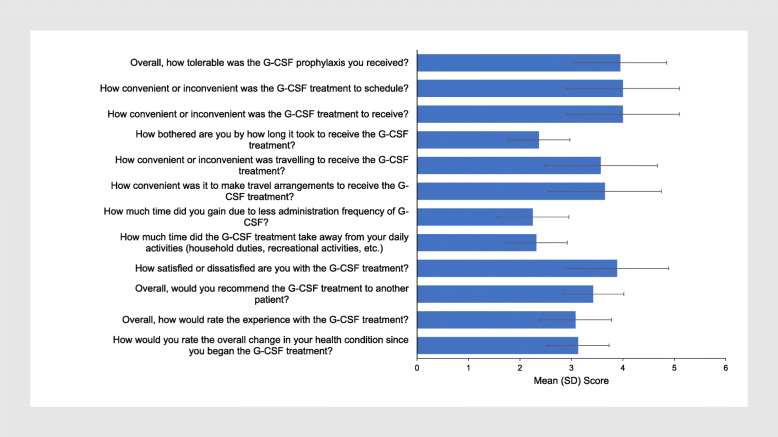
Mean relevancy scores for each item in the initial SEQ-G-CSF item relevance questionnaire (response scale: 1=not important, 2=slightly important, 3=moderately important, 4=very important, 5=extremely important). The question “Did you take the most recent prescribed G-CSF treatment?” is not scored, as it was specifically designed to determine how many patients were non-adherent to their recent G-CSF treatment

Mean relevancy scores for each item in the initial SEQ-G-CSF item relevance questionnaire are shown in Fig. 2. Based on participant feedback after round 1 of focus group discussions, some items were revised. The original and alternative items were then reviewed in round 2 of discussions. Finally, all iterations (original item and alternative item) were reviewed in round 3 of discussions. The final version of each item was based on the summary of all feedback received from participants in rounds 1, 2, and 3; item-level feedback is presented in Table 4.

**Table 4:** SEQ-G-CSF Item History

Version 1 Instrument	Revisions	Rationale for Change
Title: Satisfaction and Experience Questionnaire Core (SEQ-CORE) Granulocyte Colony-Stimulating Factor (G-CSF)	No change	• Include version number in title.
Instructions: Please read each question and select one response regarding the most recent G-CSF session, if you are currently taking G-CSF.	Revised	• Patients were not familiar with the term G-CSF. Recommend explaining what G-CSF stands for, methods of administration, and time frame (referring to the most recent treatment) for answering the questions.
Item 1 Overall, how tolerable was the G-CSF prophylaxis you received?	Revised	• Patients preferred direct and personal language. • Patients preferred “preventive” rather than “prophylaxis.” • Add reference to time frame, referring to the most recent treatment. • Revise response categories to match concept. For example, “tolerate very poorly,” “tolerate poorly,” etc. • Change chronological order: renumbered as item 2. • Recommend replacing throughout “G-CSF treatment” to “preventive treatment.”
Item 2 How convenient or inconvenient was the G-CSF treatment to schedule?	No change	• Add reference to time frame, referring to the most recent treatment. • Change response option 3 to “A little inconvenient” for ease of scoring. • Change chronological order: renumbered as Item 3.
Item 3 How convenient or inconvenient was the G-CSF treatment to receive?	Revised	• Patients preferred rewording the item for easier readability. • Add reference to time frame, referring to the most recent treatment. • Change response option 3 to “A little inconvenient” for ease of scoring. • Change chronological order: renumbered as Item 6.
Item 4 How bothered are you by how long it took to receive the G-CSF treatment?	Revised	• Patients preferred rewording the item for easier readability. • Add reference to time frame, referring to the most recent treatment. • Recommend revising to 5-point Likert scale to match other items. • Change chronological order: renumbered as Item 7.
Item 5 How convenient or inconvenient was travelling to receive the G-CSF treatment?	Revised	• Patients recommended wording changes. • Add reference to time frame, referring to the most recent treatment. • Change response option 3 to “A little inconvenient” for ease of scoring.
Item 6 How convenient was it to make travel arrangements to receive the G-CSF treatment?	Revised	• Add reference to time frame, referring to the most recent treatment. • Change response option 3 to “A little inconvenient” for ease of scoring. • Change chronological order: renumbered as Item 4.
Item 7 How much time did you gain due to less administration frequency of G-CSF?	Revised	• Patients had difficulty understanding the question/item concept. • Omit from final SEQ-G-CSF.
Item 8 How much time did the G-CSF treatment take away from your daily activities (household duties, recreational activities, etc.)?	Revised	• Add reference to time frame, referring to the most recent treatment. • Revise to 5-point Likert scale to match other items. • Change chronological order: renumbered as Item 9.
Item 9 Did you take the most recent prescribed G-CSF treatment?	Revised	• Recommend adding a clarifying statement with “No” response. • Yes (Skip to question 2) • No (Continue to question 1a) • Change chronological order: renumbered as Item 1.
Item 9a If “No” to Question 9, why did you miss the most recent G-CSF treatment?	Revised	• Add reference to time frame, referring to the most recent treatment. • Remove skip statement as it is already included in Item 1. • Remove open-ended option. • Patients provided response options. • Change chronological order: renumbered as Item 1a.
Item 9b If “No” to Question 9, how concerned are you that you missed the G-CSF treatment?	Revised	• Remove skip statement as it is already included in Item 1. • Add reference to time frame, referring to the most recent treatment. • Revise to 5-point Likert scale to match other items. • Change chronological order: renumbered as Item 1b.
Item 10 How satisfied or dissatisfied are you with the G-CSF treatment?	Revised	• Recommend adding “overall” to the item to distinguish from “most recent” items. • Change response option 3 to “A little dissatisfied” for ease of scoring.
Item 11 Overall, would you recommend the G-CSF treatment to another patient?	Revised	• Recommend replacing “G-CSF treatment” with “method of administration”. • Change chronological order: renumbered as Item 13.
Item 12 Overall, how would you rate the experience with the G-CSF treatment?	Revised	• Revise to 5-point Likert scale to match other items. • Change chronological order: renumbered as Item 11.
Item 13 How would you rate the overall change in your health condition since you began the G-CSF treatment?	Revised	• Revise to 5-point Likert scale to match other items. • Change chronological order: renumbered as Item 12.
	New	• Add new item (Item 8) to capture “satisfaction” related to time spent receiving treatment.

Except for the introductory text, the G-CSF abbreviation was removed from the final SEQ-G-CSF

### Final SEQ-G-CSF and revised conceptual framework

**Fig. 3:**
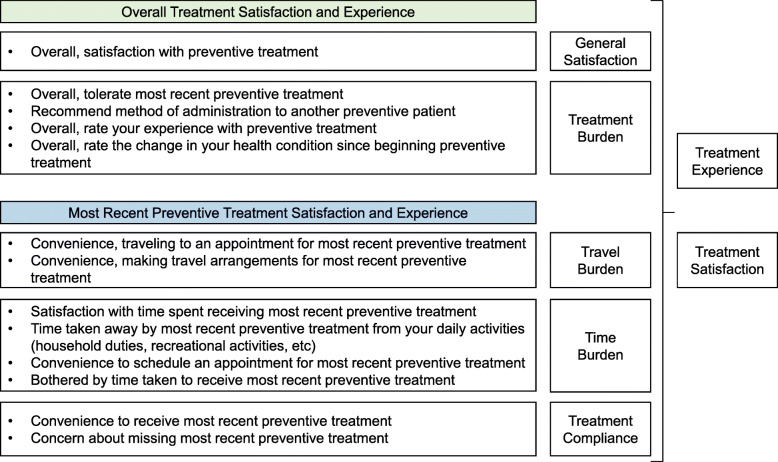
Final conceptual framework

The final SEQ-G-CSF comprises 13 items, including two items (1 and 2 in the final version) that are not scored. The conceptual framework was revised to link individual items to hypothesized PRO domains and overall summary scores (as applicable) based on findings from the focus group discussions. The final conceptual framework includes five domains related to general satisfaction (one item), treatment burden (four items), travel burden (two items), time burden (four items), and treatment compliance (two items) (Fig. 3).

## Discussion

The SEQ-G-CSF was developed and tested using a systematic, iterative process to confirm content validity based on accepted best practices and guidance for PRO development [24–27]. The SEQ-G-CSF is the first PRO instrument designed specifically to measure patients’ satisfaction and experience with different G-CSF prophylaxis options. Its inclusion in research studies, as a part of a comprehensive evaluation of different treatment options, would add important information to support patient and clinician decision making.

Several treatment satisfaction instruments were available prior to development of the SEQ-G-CSF, including those that assess satisfaction with medication delivery via injections [32–34] or satisfaction with treatment in general [23, 35, 36]. None, however, assess satisfaction and experience with prophylactic treatments administered via an injection or a device. The SEQ-G-CSF fills this gap by including five domains related to general satisfaction, treatment burden, travel burden, time burden, and treatment compliance.

The economic burden of clinic visits to receive G-CSF therapy after chemotherapy is substantial [37]. In addition, these clinic visits may be logistically challenging, requiring patients to reschedule their daily activities, arrange transportation and family care, and be absent from work [37–41]. Given these challenges, receiving pegfilgrastim 24 h after chemotherapy or multiple administrations of filgrastim every chemotherapy cycle may lead to suboptimal administration and loss of treatment benefit, ie, increased risk of FN [42, 43]. Survey results from 151 physicians who prescribed pegfilgrastim the same day as chemotherapy (suboptimal administration) provided insights into patient burden associated with optimal use. When asked to consider the importance of various patient or caregiver preferences when deciding to administer pegfilgrastim the same day as chemotherapy, most physicians considered travel distance, method or availability of transportation, understanding of and/or compliance with instructions for preparation and administration, and patient/caregiver request to avoid additional copays/office visits as moderately or very important [40].

In our study, patients most commonly rated G-CSF effectiveness, convenience and benefits of the OBI, and relationships with healthcare providers as positive experiences, whereas side effects, undergoing additional treatment, injection discomfort, and sleep interruption were the most common negative experiences. Surprisingly, economic burden did not factor into patients’ satisfaction with G-CSF treatment. The SEQ-G-CSF can help clinicians assess the quality of G-CSF treatment in terms of process, which is one of the three pillars of healthcare quality [44]. In addition, it will help to anticipate patients’ needs—according to the Institute of Medicine, patient-centered healthcare should be an integral part of the healthcare system [45]. Despite the potential response burden of completing such a measure in oncology trials, patients with cancer may not perceive the experience as overly burdensome [46].

Our study has several limitations. The overall small sample size makes it difficult to generalize patients’ treatment experience and satisfaction to the broader oncology patient population. In addition, there was heterogeneity in G-CSF prophylaxis experiences; participants received several types of G-CSF prophylaxis with varying modes of delivery, frequency, and duration of treatment. Furthermore, in the event of time limitations during each focus group discussion, the interviewer prioritized the review of SEQ-G-CSF items for which participants had proposed changes in prior discussions.

## Conclusions

With the availability of different options for administering G-CSF prophylaxis, it is important to support decision making with patients’ perspectives of and satisfaction with their treatment experience. The SEQ-G-CSF is a novel PRO measure that quantifies a patient’s experience and satisfaction with different G-CSF options, using 13 easy-to-understand items. While additional testing is required, this study provides evidence of content validity. The use of SEQ-G-CSF in further studies may provide an opportunity for further psychometric testing, including item reduction, scoring, reliability, and construct validity assessment, which is necessary to support the use of the SEQ-G-CSF in scientific studies and clinical practice. After additional testing, the SEQ-G-CSF may be a useful addition to clinical trials, observational studies, and clinical practice.

## Supplementary Information

**Additional file 1.**

## Data Availability

Qualified researchers may request data from Amgen clinical studies. Complete details are available at http://www.amgen.com/datasharing.

## Abbreviations

G-CSF: Granulocyte colony-stimulating factor
NHL: Non-Hodgkin lymphoma
OBI: On-body injector
PRO: Patient-reported outcome
SEQ-G-CSF: Satisfaction and Experience Questionnaire for G-CSF
