# Supplementary material for: Development and content validation of the Satisfaction and Experience Questionnaire for Granulocyte Colony-Stimulating Factor (SEQ-G-CSF)
Source: J Patient Rep Outcomes. 2021 Jan 18;5:10. doi: 10.1186/s41687-020-00277-8 (PMC7813942; doi:10.1186/s41687-020-00277-8)
Supplement: Supplementary file 1 — Additional file 1. [file 41687_2020_277_MOESM1_ESM.docx]

# Supplementary materials

## SEQ Module 1: Sociodemographic Form

**Participant ID: __________ Participant Initials: _______** **Date: ____/____/____**

**DD MM YYYY**

***Instructions:*** Please read each question and select your response.

| 1. | What is your age? |
| --- | --- |
|  | _________________ Years |
| 2. | What is your gender? |
|  | 🞏 Male  🞏 Female |
| 3. | What is your ethnic background? |
|  | 🞏 Hispanic or Latino  🞏 Not Hispanic or Latino |
| 4. | What is your racial background? *(Please check all that apply)* |
|  | 🞏 American Indian or Alaska Native  🞏 Asian  🞏 Black or African American  🞏 Native Hawaiian or Pacific Islander  🞏 White  🞏 Other race (*Please specify*): _________________________________ |
| 5. | How would you describe your employment status? |
|  | 🞏 Employed, full-time  🞏 Employed, part-time  🞏 Homemaker  🞏 Student  🞏 Unemployed  🞏 Retired  🞏 Disabled  🞏 Other (*Please specify*):____________________________________ |
| 6. | What is the highest level of education you have completed? |
|  | 🞏 Elementary/primary school  🞏 Secondary/high school/GED  🞏 Some college or post-high school education or training  🞏 College degree  🞏 Postgraduate degree  🞏 Other (Please specify): __________________________________________ |
| 7. | What is your insurance status? |
|  | 🞏 Insured, minimal out-of-pocket costs  🞏 Insured, significant out-of-pocket costs  🞏 Not insured |

## SEQ Module 2: Medical History Form (G-CSF Version)

**Participant ID: __________ Participant Initials: _______ Date: ____/____/____**

**DD MM YYYY**

| 1. | What type of cancer do you have? |
| --- | --- |
|  | 🞏 Lung Cancer  🞏 Breast cancer  🞏 Prostate cancer  🞏 Non-Hodgkin's Lymphoma (NHL) |
| 2. | What month/year did you receive the cancer diagnosis? |
|  | _________/_________  Month Year |
| 3. | Has your cancer spread to another region or organ? |
|  | 🞏 Yes  🞏 No |
| 4. | Are you currently receiving chemotherapy treatment? |
|  | 🞏 Yes  🞏 No |
| 5. | Have you recently stopped chemotherapy treatment? |
|  | 🞏 Yes  🞏 No |
| 6. | What date was your last chemotherapy treatment? |
|  | _________/_________  Month Year |
| 7. | Have you *recently received OR are you currently receiving* treatment with Granulocyte Colony Stimulating Factor (G-CSF) as prophylaxis to minimize your risk of Febrile Neutropenia/infection from chemotherapy? |
|  | 🞏 Yes, recently received and completed G-CSF prophylaxis  🞏 Yes, currently receiving G-CSF prophylaxis |
| 8. | Can you recall the name of the G-CSF prophylaxis you have received? |
|  | 🞏 Pegfilgrastim (Examples: Neulasta, or Fulphila)  🞏 Filgrastim (Examples: Neupogen, Zarxio, Granix, or Nivestym)  🞏 Others (*Please specify*): ___________________ |
| 9. | What date was your last G-CSF prophylaxis? |
|  | _________/_________  Month Year |
| 10. | How is/was your G-CSF prophylaxis administered? (*select only one answer*) |
|  | 🞏 Intravenous (IV)  🞏 Subcutaneous (SC)  🞏 On-body Injector (OBI) |
| 11. | Have you ever used an on-body injector for G-CSF treatment? |
|  | 🞏 Yes  🞏 No |
| 12. | If you have stopped using G-CSF prophylaxis, what was the reason(s) for discontinued use? |
|  | ______________________________________________________________________ |
| 13. | Have you been diagnosed by a medical doctor with any other serious health conditions besides your cancer? |
|  | 🞏 No other health condition  🞏 Kidney disease  🞏 Liver disease  🞏 Cardiovascular disease  🞏 Other serious health condition (*Please specify*): _______________________________ |
| 14. | In general, how would you say your health was within the past week? |
|  | 🞏 Excellent  🞏 Very good  🞏 Good  🞏 Fair  🞏 Poor |

## SEQ Module 3: G-CSF Healthcare Characteristics Questionnaire

**Participant ID: __________ Participant Initials: _______ Date: ____/____/____**

**DD MM YYYY**

***Instruction:*** Please read each question and select your response.

| 1. | How long did it take you to travel one way to receive your treatment? | | | | | | | | | | | |
| --- | --- | --- | --- | --- | --- | --- | --- | --- | --- | --- | --- | --- |
|  | _____ Hours _____Minutes | | | | | | | | | | | |
| 2. | If you made multiple visits since your most recent chemotherapy, please take the average. Your best estimate is fine. If you had no travel, please enter “0” hours, “0” minutes | | | | | | | | | | | |
|  | _________/_________  Hours/Minutes | | | | | | | | | | | |
| 3. | How do you usually get travel to the site/clinic to receive your chemotherapy treatment? | | | | | | | | | | | |
|  | 🞏 No travel was required  🞏 I drove myself  🞏 Someone else drove me  🞏 Taxi  🞏 Public transportation (bus, train etc.)  🞏 Other (e.g., walk) _______________________ | | | | | | | | | | | |
| 4. | How often did you travel to receive your cancer treatment? | | | | | | | | | | | |
|  | 🞏 Less often than once per month  🞏 Once per month  🞏 2-3 times per month  🞏1 day per week  🞏 2 days per week  🞏 3 or more days per week  🞏 Other ________________________ | | | | | | | | | | | |
| 5. | Did you have someone to drive you/take you to receive your G-CSF prophylaxis? | | | | | | | | | | | |
|  | 🞏 No, self-support  🞏 Yes, an unpaid caregiver (family, friend, etc.)  🞏 Yes, a paid care provider (helper, personal care assistant)  🞏 Yes, clinic's arrangement  🞏 Other | | | | | | | | | | | |
| 6. | Did you have someone to accompany you to doctor's visits related to your G-CSF prophylaxis? | | | | | | | | | | | |
|  | 🞏 Yes  🞏 No | | | | | | | | | | | |
| 7. | When you started G-CSF prophylaxis, did your cancer therapy team clearly explain about this treatment and its options? | | | | | | | | | | | |
|  | 🞏 Yes  🞏 No | | | | | | | | | | | |
| 8. | In the last 6 months, did your cancer therapy team tell you what the next steps in your chemotherapy would be? | | | | | | | | | | | |
|  | 🞏 Yes  🞏 No  🞏 Other | | | | | | | | | | | |
| 9. | In the last 6 months, did your cancer therapy team explain what G-CSF was for in a way that was easy to understand (if a medicine was prescribed that you had not taken before)? | | | | | | | | | | | |
|  | 🞏 Yes  🞏 No | | | | | | | | | | | |
| 10. | After it was decided to have G-CSF prophylaxis, did your cancer therapy team encourage you to contact them with your questions about your G-CSF prophylaxis? | | | | | | | | | | | |
|  | 🞏 Yes  🞏 No | | | | | | | | | | | |
| 11. | Did your cancer therapy team discuss possible side effects of the G-CSF Chemotherapy? | | | | | | | | | | | |
|  | 🞏 Detailed explanation  🞏 Written  🞏 No information  🞏 Other ________________________ | | | | | | | | | | | |
| 12. | How much time did you spend in the office/clinic to receive your cancer treatment? | | | | | | | | | | | |
|  | _________/_________  Hours/Minutes | | | | | | | | | | | |
| 13. | Have you experienced a benefit from the cancer treatment? | | | | | | | | | | | |
|  | 🞏 Yes, much benefit  🞏Yes, a little benefit  🞏 No benefit  🞏 Don’t know | | | | | | | | | | | |
| 14. | If yes to Question 13, were you able to tolerate the cancer treatment given the side effects? | | | | | | | | | | | |
|  | 🞏 Yes  🞏 No  🞏 Other | | | | | | | | | | | |
| 15. | Did you have to pay for any of the following to receive your G-CSF prophylaxis? | | | | | | | | | | | |
|  | 🞏 Gas  🞏 Fares to and from the clinic (e.g., Taxi, bus, train)  🞏 Fares for a return trip home (e.g., Flight, taxi, bus, train)  🞏 Parking  🞏 Car Rental  🞏 Temporary lodging  🞏 Other (*Please specify*) __________ | | | | | | | | | | | |
| 16. | Have you experienced side effects from the cancer treatment? | | | | | | | | | | | |
|  | 🞏 Yes, many side effects  🞏 Yes, a few side effects  🞏 No, no side effects  🞏 I don’t know | | | | | | | | | | | |
| 17. | How much did you have to pay for these items for every clinic visit to receive G-CSF prophylaxis? | | | | | | | | | | | |
|  | 🞏 Gas: $_________  🞏 Fares to and from the clinic (e.g., Taxi, bus, train): $__________  🞏 Fares for a return trip back home (e.g., Flight, taxi, bus, train): $__________  🞏 Parking: $__________  🞏 Car Rental: $__________  🞏 Temporary lodging: $__________  🞏 Other (Please specify) __________: $ __________ | | | | | | | | | | | |
| 18. | How much did you have to pay out-of-pocket for your G-CSF prophylaxis? | | | | | | | | | | | |
|  | 🞏 No out-of-pocket cost, 100% covered by insurance  🞏 Treatment: $________ | | | | | | | | | | | |
| 19. | Using any number from 0 to 10, where 0 is the worst cancer therapy team possible and 10 is the best cancer therapy team possible, what number would you use to rate your cancer therapy team over the last 6 months? | | | | | | | | | | | |
|  | 0 | 1 | | 2 | 3 | 4 | 5 | 6 | 7 | 8 | 9 | 10 |
| Worst cancer therapy  team possible | | | Mid-point | | | | | | | | | Best cancer  therapy team possible |
| 20. | How often were the office visits scheduled at times that were convenient for you during the course of your chemotherapy treatment? | | | | | | | | | | | |
|  | 🞏 All the time  🞏 Sometimes  🞏 Never | | | | | | | | | | | |

## Initial SEQ-G-CSF Item Relevancy Questionnaire

**Participant ID: __________ Participant Initials: _______ Date: ____/____/____**

Please rate how important the items on the questionnaire about your experiences with cancer treatment on a scale of 1 to 5, (“not important” to 5 “extremely important”). Please rate how important each question on the SEQ is to you.

|  | **Not Important** | **Slightly Important** | **Moderately Important** | **Very Important** | **Extremely Important** |
| --- | --- | --- | --- | --- | --- |
| 1. Overall, how tolerable was the G-CSF prophylaxis you received? | 1 | 2 | 3 | 4 | 5 |
| 1. How convenient or inconvenient was the G-CSF treatment to schedule? | 1 | 2 | 3 | 4 | 5 |
| 1. How convenient or inconvenient was the G-CSF treatment to receive? | 1 | 2 | 3 | 4 | 5 |
| 1. How bothered are you by how long it took to receive the G-CSF treatment? | 1 | 2 | 3 | 4 | 5 |
| 1. How convenient or inconvenient was travelling to receive the G-CSF treatment? | 1 | 2 | 3 | 4 | 5 |
| 1. How convenient was it to make travel arrangements to receive the G-CSF treatment? | 1 | 2 | 3 | 4 | 5 |
| 1. How much time did you gain due to less administration frequency of G-CSF? | 1 | 2 | 3 | 4 | 5 |
| 1. How much time did the G-CSF treatment take away from your daily activities (household duties, recreational activities, etc.) | 1 | 2 | 3 | 4 | 5 |
| 1. Did you take the most recent prescribed G-CSF treatment? | 1 | 2 | 3 | 4 | 5 |
| **9a.** If “No” to Question 9, why did you miss the most recent G-CSF treatment | 1 | 2 | 3 | 4 | 5 |
| **9b**. If “No” to Question 9, how concerned are you that you missed the G‑CSF treatment? | 1 | 2 | 3 | 4 | 5 |
| 1. How satisfied or dissatisfied are you with the G-CSF treatment? | 1 | 2 | 3 | 4 | 5 |
| 1. Overall, would you recommend the G-CSF treatment to another patient? | 1 | 2 | 3 | 4 | 5 |
| 1. Overall, how would you rate the experience with the G-CSF treatment? | 1 | 2 | 3 | 4 | 5 |
| 1. How would you rate the overall change in your health condition since you began the G-CSF treatment? | 1 | 2 | 3 | 4 | 5 |
